# Three-stage interpretability analysis of influenza virus and meteorological correlation in Jiuquan City, 2016–2025: SARIMAX + TreeSHAP

**DOI:** 10.3389/fcimb.2026.1791100

**Published:** 2026-06-10

**Authors:** Biao Wang, Xia Han, Maoxing Dong, Hui Zhang, Hong Shi, Huan Wei, Miao Wang, Xiaoshu Zhang, Shu Liang, Congshan Xu

**Affiliations:** 1 Gansu Provincial Center for Disease Control and Prevention, Lanzhou, China; 2 Public Health School, Gansu University of Chinese Medicine, Lanzhou, China; 3 Jiuquan Center for Disease Control and Prevention, Jiuquan, China; 4 Lanzhou Center for Disease Control and Prevention, Lanzhou, China

**Keywords:** COVID-19 pandemic, influenza, meteorological factors, non-pharmaceutical interventions, time-series dynamics

## Abstract

**Objective:**

The COVID-19 pandemic has profoundly altered global influenza circulation. This study aimed to investigate the impact of meteorological factors on influenza transmission in Jiuquan, China, during three distinct phases: before, during, and after the COVID-19 pandemic.

**Methods:**

Weekly influenza surveillance and concurrent weather data were modeled using seasonally adjusted SARIMAX to capture temporal and seasonal structures. An XGBoost surrogate was trained to emulate the SARIMAX outputs, and TreeSHAP was applied to decompose and quantify the relative contributions of the environmental variables.

**Results:**

2016-2019: Influenza followed the northern hemisphere winter-spring pattern, with A/H3N2 being predominant. 2020: COVID-19 non-pharmaceutical interventions suppressed transmission; positivity rates decreased sharply, and a 78-week “low circulation” persisted. Post-mitigation relaxation: 2023–24 winter resurgence. Weeks with positivity >10% increased from approximately seven pre-pandemic to approximately 11 post-pandemic. Overall positivity differed across phases (pre:25.29%; pandemic:12.07%; post:12.42%; P<0.001). SARIMAX explained moderate variance pre-COVID (R^2^=0.5189), little during/after pandemic (R^2^=0.0373, 0.0671). TreeSHAP surrogate achieved high fidelity (R^2^=0.8121-0.8628). Temperature was dominant across phases: low temperatures were linked to higher positivity, and high temperatures suppressed it. Pandemic phase: Wind speed, humidity, and pressure gained importance. Post-pandemic: temperature differentials strengthened, wind and pressure distributions’ influence “broadened”.

**Conclusion:**

During COVID-19, NPIs drastically suppressed influenza transmission in Jiuquan, disrupting its winter-spring cycle and causing prolonged “low circulation”. Following relaxation, immunity debt fueled stronger and longer seasonal resurgences, with subtype niche shifts; influenza rebounded but not to pre-pandemic levels after relaxation. Stable temperature effects and shifting roles of wind, pressure, and thermal gradients may reflect environmental-behavioral transmission restructuring, highlighting region-specific, immunity- and seasonality-informed control strategies.

## 1 Introduction

Influenza is an acute respiratory illness caused by the influenza virus (Nypaver et al., 2021), characterized by rapid transmission, high mutability, and universal susceptibility, rendering it prone to large-scale epidemics and outbreaks (Forleo-Neto et al., 2003). As a significant global public health threat, influenza affects approximately 10% of adults and 20% of children annually, resulting in nearly 1 billion infections, 3–5 million hospitalizations, and 290,000–650,000 respiratory deaths each year (Qi et al., 2020; Paget et al., 2023; Jain et al., 2025). In China alone, seasonal influenza contributes to an estimated 88,000 excess respiratory deaths annually (Fu et al., 2024). Meteorological factors—including temperature, humidity, and atmospheric pressure—modulate the stability and transmissibility of respiratory viruses and may impair host immune defenses, collectively driving the heightened viral activity observed during autumn and winter (Moriyama et al., 2020). The COVID-19 pandemic emerged precisely during the peak transmission season for respiratory viruses such as influenza (Leuzinger et al., 2020). In response, governments worldwide implemented a range of non-pharmaceutical interventions (NPIs)—including lockdowns, travel restrictions, social distancing mandates, mandatory mask-wearing, and closures of schools and commercial venues—to suppress SARS-CoV-2 transmission. Although these measures proved effective in controlling COVID-19, they simultaneously and profoundly altered the transmission dynamics of other respiratory pathogens, including influenza (Leuzinger et al., 2021; Tempia et al., 2021; Park et al., 2021; Chow et al., 2023). Notably, the transmission patterns of seasonal influenza in northwestern China have undergone substantial shifts (Wang et al., 2025). Emerging evidence indicates that influenza virus detection rates have trended upward in the post-pandemic period, suggesting potential changes in viral ecology and transmission dynamics (World Health Organization, 2023). These developments warrant rigorous investigation to clarify their underlying drivers and public health implications. Given China’s vast territory, diverse climate zones, and enormous population, influenza activity exhibits considerable regional and seasonal heterogeneity (Li et al., 2019), with temperate regions displaying particularly pronounced seasonal epidemic patterns (Du et al., 2022). Jiuquan City is situated in the western Hexi Corridor of northwestern Gansu Province (39°43′N, 98°30′E), at an elevation of approximately 1,000–1,600 m above sea level. Positioned along the transitional zone between the northern foothills of the Qilian Mountains and the Gobi Desert, the city features a temperate continental arid to semi-arid climate distinguished by scarce precipitation, pronounced diurnal temperature variation, abundant solar radiation, and frequent springtime sandstorms. The interplay of these topographic and climatic characteristics shapes both viral survival conditions and human exposure patterns, establishing Jiuquan as an ecologically representative site for investigating the relationship between meteorological factors and influenza transmission.

Since the global emergence of COVID-19 in late 2019, NPI implementation has substantially altered human environmental exposure and interpersonal contact behaviors (Huang et al., 2021), potentially reconfiguring the relationship between environmental conditions and influenza infection risk. Reassessing these dynamic associations in the post-pandemic context is therefore both timely and necessary.

This study aimed to elucidate the complex interplay between environmental factors and influenza transmission dynamics in Jiuquan—a representative city of China’s arid and semi-arid northwest—by characterizing epidemiological differences across the pre-pandemic, pandemic, and post-pandemic phases. Through a structured phased analysis, we quantified the evolution of meteorological influences on influenza transmission before and after NPI implementation and assessed whether the underlying environmental driving structure has undergone substantive restructuring.

Methodologically, this study employed the Seasonal Autoregressive Integrated Moving Average with Exogenous Variables (SARIMAX) model to capture the temporal dependence and seasonality inherent in weekly influenza positivity rates. As a well-established time-series analytical framework, SARIMAX is widely applied in long-term infectious disease surveillance owing to its conceptual transparency, modeling flexibility, and demonstrated reliability (Liu et al., 2020; Qi et al., 2020; Qiu et al., 2021). To address its inherent interpretability constraints, this study innovatively integrated the SARIMAX model with a TreeSHAP surrogate model, enabling a granular decomposition of the relative contributions and directional effects of seven meteorological factors: air temperature, relative humidity, wind speed, sunshine duration, atmospheric pressure, precipitation, and daily mean temperature range. By applying this integrated framework across three distinct epidemiological phases—pre-pandemic, pandemic, and post-pandemic—this study provides a quantitative assessment of how environmental drivers evolved in response to NPI interventions, offering a methodological framework that harmonizes predictive capacity with mechanistic interpretability for post-pandemic influenza–environment research.

## 2 Materials and methods

### 2.1 Influenza surveillance data

The data for this study were obtained from influenza surveillance data collected in Jiuquan City from January 1, 2016, to December 31, 2025, within China’s Influenza Surveillance Information System. The data included daily influenza virus testing volumes, detection rates, and predominant circulating subtypes in Jiuquan City (influenza viruses classified according to the WHO nomenclature: influenza A subtypes A(H1N1)pdm09 and A(H3N2), and influenza B Victoria and Yamagata lineages). For brevity, these are hereafter referred to as A/H1N1, A/H3N2, B/Victoria, and B/Yamagata. The positivity rate for each influenza subtype was calculated using the following formula:


$$\text{Positive Rate subtype}\left(\%\right)=\frac{\text{Npositive, subtype}}{/\text{Ntested}}\times 100\%$$

To analyze the impact of meteorological factors on the transmission of influenza subtypes, we divided the study period into three distinct phases based on the timeline of the COVID-19 pandemic in Jiuquan, China.

- Pre-pandemic Period (January 1, 2016, to December 31, 2019): This refers to the period before SARS-CoV-2 was detected in Jiuquan, China, during which no non-pharmaceutical interventions were implemented.
- Pandemic Period (January 1, 2020, to December 31, 2022): Public health interventions were implemented during this phase.
- Post-Pandemic Period (January 1, 2023, to December 31, 2025): Zero-COVID policies were relaxed during this phase.

### 2.2 Meteorological data

Meteorological data are publicly accessible after registration on the official website of the National Meteorological Science Data Center (data.cma.cn/). The meteorological factors included the weekly average temperature (°C), weekly average temperature difference (°C), weekly average air pressure (hPa), weekly average precipitation (mm), weekly average relative humidity (%), weekly average sunshine duration (h), and weekly average wind speed (m/s).

### 2.3 Data preprocessing

Data processing and statistical analyses were performed using the R4.5.3 software. Normality was assessed using the Shapiro-Wilk test, and Levene’s test was applied to evaluate the homogeneity of variance. When the data were normally distributed with equal variances, ANOVA was used; when normally distributed but with unequal variances, Welch’s ANOVA was employed. The Kruskal-Wallis test was performed for continuous variables that did not meet the normality assumption. *Post-hoc* pairwise comparisons used Dunn’s test for normally distributed data and the Mann-Whitney U test for non-normally distributed data. All pairwise comparisons were performed using the Bonferroni correction for multiple comparisons. Categorical variables were compared between groups using the chi-square or Fisher’s exact tests. Statistical significance was set at P< 0.05. Significance levels are denoted as follows: *** p<0.001, ** p<0.01, * p<0.05, ns p≥0.05.

### 2.4 SARIMAX time series modeling

The Seasonally Autoregressive Integrated Moving Average model (SARIMAX) was adopted as the base forecasting model, with the following mathematical expression:


$$\phi \left(\text{B}\right)\Phi \left({\text{B}}^{\text{s}}\right){\left(1-\text{B}\right)}^{\text{d}}{\left(1-{\text{B}}^{\text{s}}\right)}^{\text{D}}{\text{y}}_{\text{t}}=\theta \left(\text{B}\right)\Theta \left({\text{B}}^{\text{s}}\right)\epsilon_{\text{t}}+\beta {\text{X}}_{\text{t}}\phi \left(\text{B}\right)\Phi \left(\text{Bs}\right)\left(1-\text{B}\right)\text{d}\left(1-\text{Bs}\right)\text{Dyt}=\theta \left(\text{B}\right)\Theta \left(\text{Bs}\right)\epsilon \text{t}+\beta \text{Xt}$$

where *y_t_*y_t_ denotes the influenza virus positivity rate at time *t* t, *X_t_*X_t_ represents the exogenous meteorological variable matrix, *B*B signifies the lag operator, *s* s indicates the seasonal cycle (set at 52 weeks), and *ϕ(B)* ϕ(B), *Φ(B^s^)*Φ(Bs), *θ(B)*θ(B), and *Θ(B^s^)*Θ(Bs) denote non-seasonal and seasonal autoregression and moving average polynomials, respectively. *d*d and *D*D represent the order of non-seasonal and seasonal differencing, *β*β is the regression coefficient for exogenous variables, and ϵt is the white-noise error term. Model selection was performed using the auto.arima function (R package forecast v8.21), with the AICc as the selection criterion. The final model structures were ARIMA(1,0,3)(0,0,0)[52] for the pre-pandemic period, ARIMA(1,1,3)(0,0,0)[52] for the pandemic period, and ARIMA(0,1,0)(0,0,0)[52] for the post-pandemic period, with meteorological variables incorporated as external regressors in all three phases.

### 2.5 TreeSHAP proxy model construction

To enable the interpretability analysis of the SARIMAX model, an XGBoost proxy model was constructed to fit the SARIMAX predictions. An XGBoost regression model was trained using meteorological variables as input features and the SARIMAX model fitted values as the target variable. Optimal parameter combinations were determined through 5-fold cross-validation, including learning rate (η=0.1), maximum depth (max_depth=4), minimum child weight (min_child_weight=3), subsample rate (subsample=0.8), and feature sampling rate (colsample_bytree=0.8). The coefficient of determination (R^2^) between the proxy model predictions and SARIMAX fitted values was calculated as a fidelity metric to ensure that the proxy model accurately reflected the predictive behavior of the original model.

### 2.6 TreeSHAP interpretability analysis


$$\phi \text{i}=\sum_{\text{s}\subseteq \text{F}\backslash \left\{i\right\}}\frac{|\text{S}|!\left(|\text{F}|-|\text{S}|-1\right)!}{|\text{F}|!}\left[\text{f}\left(\text{S}\cup \text{i}\right)-\text{f}\left(\text{S}\right)\right]\phi \text{i}=\text{S}\subseteq \text{F}\backslash \left\{\text{i}\right\}\sum |\text{F}|!|\text{S}|!\left(|\text{F}|-|\text{S}|-1\right)!\left[\text{f}\left(\text{S}\cup \left\{i\right\}\right)-\text{f}\left(S\right)\right]$$

among these, *ϕi* ϕi represents the SHAP value for feature *i*i, *F*F denotes the full feature set, *S*S denotes the feature subset excluding feature *i*i, and *f(·)* f(·)is the model prediction function. The average absolute SHAP value for each feature was calculated as a global importance metric:


$${\text{Importance}}_{\text{i}}=\frac{1}{\text{n}}{\sum_{\text{j}=1}}^{\text{n}}|\phi_{\text{i,j}}|\text{Importancei}=\text{n}1\text{j}=1\sum \text{n}|\phi \text{i,j}|$$

Relative importance is defined as:


$${\text{Relative\_Importance}}_{\text{i}}={\sum_{k=1}}^{p}\frac{{\text{Importance}}_{\text{i}}}{{\text{Importance}}_{\text{k}}}\times 100\%\text{Relative\_Importancei}=\sum \text{k}=1\text{p Importancek Importancei}\times 100\%\;$$

### 2.7 Model validation and performance evaluation

The model performance was evaluated using a forward-chaining cross-validation approach, in which the chronological ordering of weekly time-series observations was strictly preserved across all training and validation iterations. At each step, the SARIMAX model was fitted exclusively on chronologically antecedent observations and validated on the immediately succeeding time window, thereby precluding temporal data leakage and ensuring that the model performance estimates reflected genuine out-of-sample predictive capability. The predictive accuracy was quantified using the root mean square error (RMSE) and the coefficient of determination (R^2^) as primary performance metrics.

## 3 Results

### 3.1 SARIMAX + TreeSHAP surrogates model performance

Our SARIMAX + TreeSHAP Surrogates model demonstrated relatively robust explanatory power during the early stage of the pandemic, while exhibiting limited explanatory power during the pandemic and post-pandemic periods. The R^2^ values across the three periods ranged from 0.0373 to 0.5189, with RMSE values fluctuating between 0.0833 and 0.1806. Details are provided in **Supplementary Table 1**.

### 3.2 Sensitivity analysis: model performance excluding strict NPI periods

To isolate meteorological signals from NPI-related confounding, a pooled SARIMAX model was fitted to the pre-pandemic (2016–2019) and post-pandemic (2023–2025) periods, excluding 2020–2022. The resulting ARIMA(2,0,2)(0,0,0)[52] model (AICc = −354.42) achieved R^2^ = 0.278, a 7.5-fold improvement over the pandemic-period model (R^2^ = 0.037), with TreeSHAP surrogate fidelity of 0.788. The stationary model structure (d = 0), in contrast to the non-stationary pandemic model (d = 1), suggests that the reduced meteorological associations observed during 2020–2022 reflect NPI-induced disruption rather than an inherent property of influenza dynamics. Details are provided in **Supplementary Table 2**.

### 3.3 Epidemiological dynamics of seasonal influenza in Jiuquan

**Figure 1:**
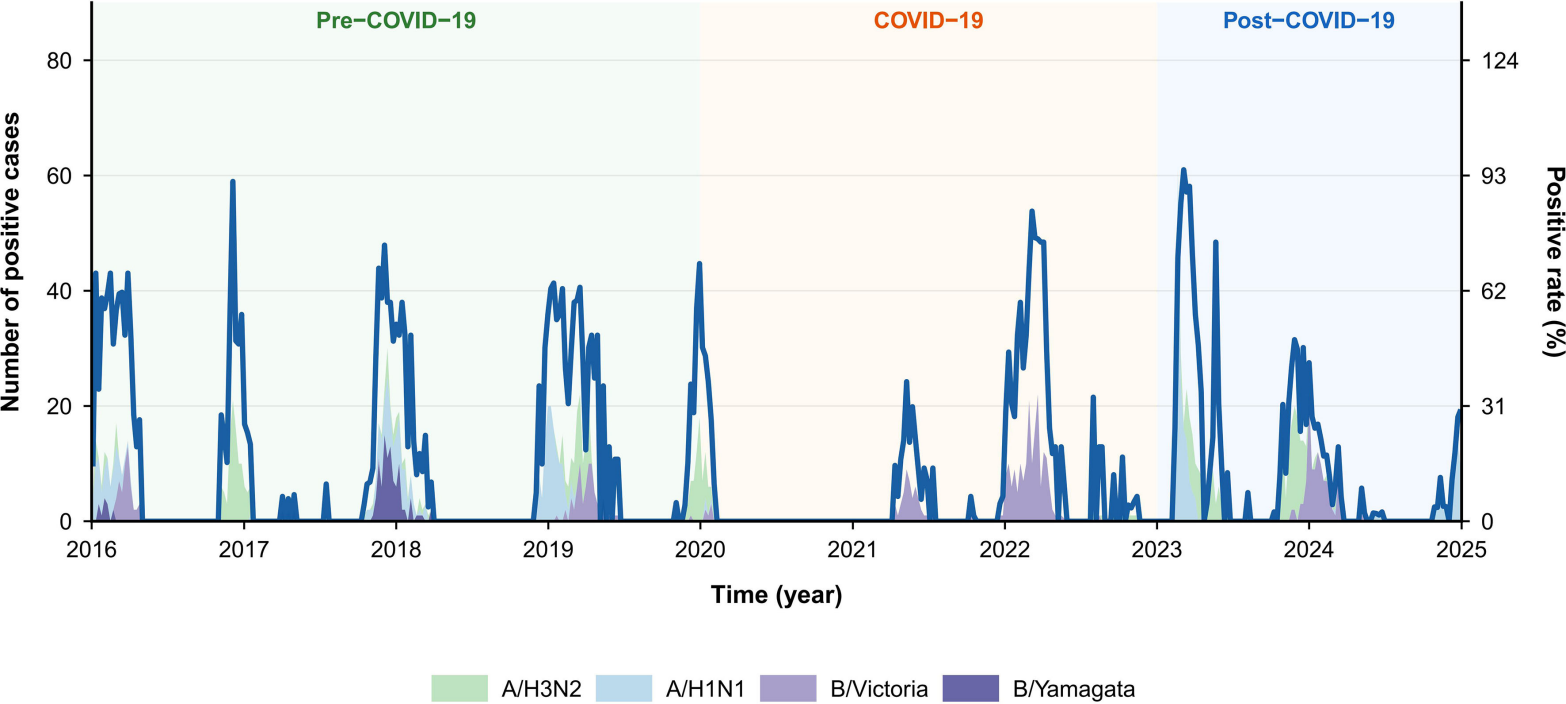
Temporal trends in the number of cases and positive rates for the influenza A subtypes and B lineage (A/H3N2, A/H1N1,B/Victoria,B/Yamagata) from 2016 to 2025.

Before the COVID-19 outbreak, influenza virus circulation in Jiuquan, China, exhibited typical seasonal patterns of the northern hemisphere, with annual peaks occurring from winter to the following spring. These outbreaks are usually dominated by a single subtype, despite the coexistence of multiple subtypes. The data revealed distinct antigenic drift among subtypes: A/H3N2 predominated in most years, B/Victoria maintained a relatively stable prevalence, and B/Yamagata was detected only sporadically. Notably, A/H1N1 exhibited intermittent outbreaks. However, during 2020–2021, all subtypes experienced epidemiological silence, consistent with the suppression of influenza transmission by non-pharmaceutical interventions for COVID-19. Weekly positivity rates plummeted to<5% starting from March 2020. No traditional winter epidemic peaks were observed for 78 consecutive weeks from April 2020 to October 2021, with weekly case counts remaining at historically low levels of 0–5 cases. During the 2021–2022 winter season, only a slight resurgence of the B/Victoria subtype was observed, whereas influenza A was almost entirely absent, suggesting potential differences in susceptibility to non-pharmaceutical interventions among subtypes. In the post-pandemic era, as national control measures were lifted and epidemic management gradually normalized, the 2023–2024 influenza season exhibited a typical, high-intensity seasonal peak occurring during the winter months from late 2023 to early 2024. The peak height of positive cases indicated a significant rebound in overall influenza activity, breaking free from the unusually suppressed state observed between 2020 and 2022. The A/H3N2 subtype once again became the dominant circulating subtype, covering the largest geographic area and accounting for the vast majority of positive cases during the season. The A/H1N1 and B/Victoria subtypes were also detected but exhibited relatively low activity levels and maintained a co-circulating pattern. Notably, the duration of positive rates exceeding 10% extended from the pre-pandemic average of seven weeks to 11 weeks, suggesting that the “immunological debt” effect led to the accumulation of susceptible populations. The peak pattern of the 2024–2025 winter season further evolved, with alternating dominance between A/H3N2 and A/H1N1, indicating the ongoing restructuring of subtype niches. B/Yamagata failed to establish effective circulation during the post-pandemic period. As shown in **Figure 1**.

**Figure 2:**
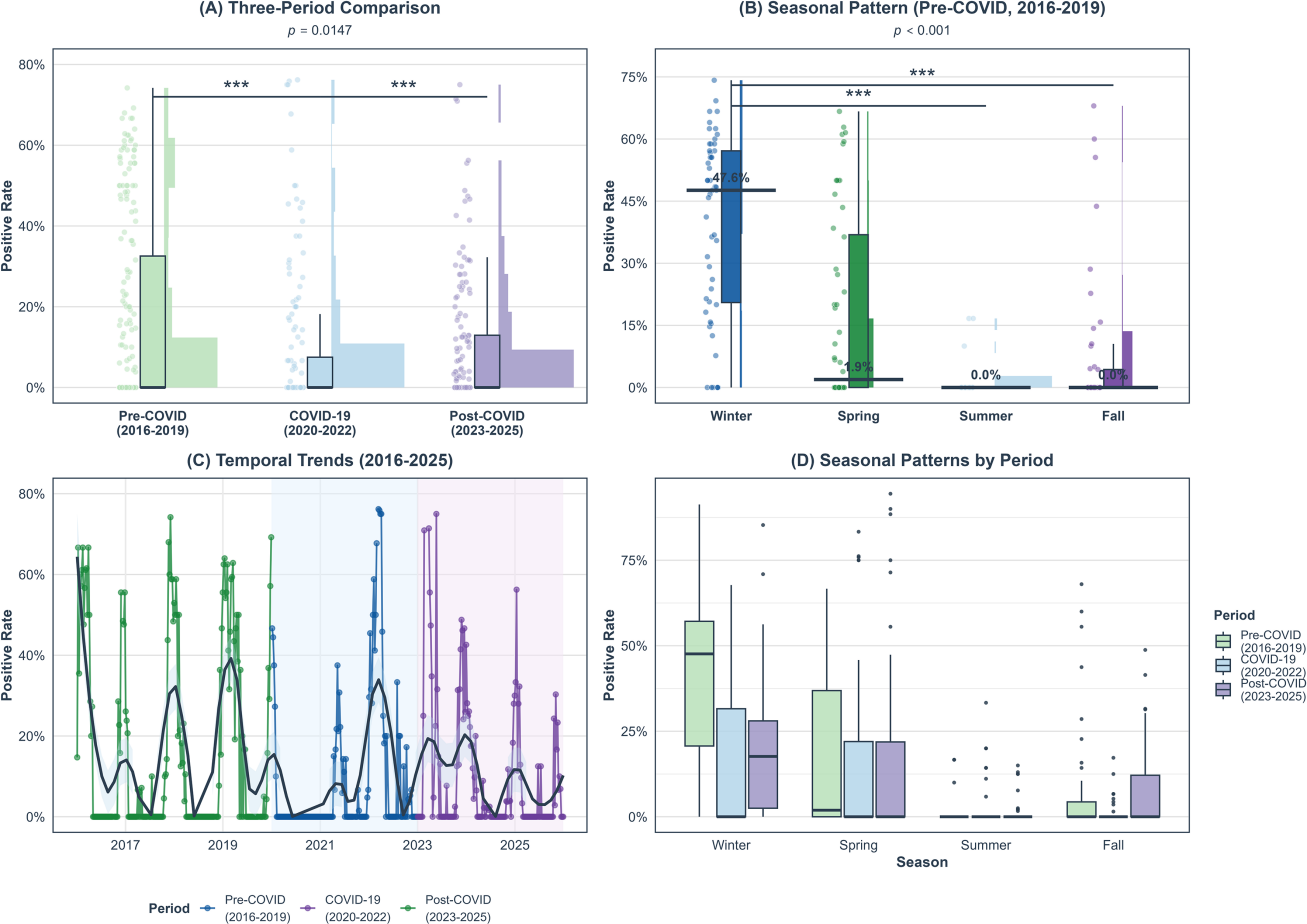
Spatiotemporal patterns of influenza positive rates from 2016 to 2025, incorporating **(A)** three-period comparisons, **(B)** Pre-COVID seasonal variation, **(C)** long-term temporal trends across pandemic phases(green: Pre-COVID-19;blue: COVID-19; purple: Post-COVID-19); black line indicates smoothed overall trend. **(D)** period-stratified seasonal patterns with statistical significance.* Indicates statistical significance (p<0:05), *** Indicates statistical significance (p<0:001), n.s. Indicates statistical significance.

This study compared the distribution and temporal changes in the weekly influenza positivity rates during three periods and across seasons from 2016 to 2025. The results showed significant differences in positivity rates across the three study periods (p=0.0147). As shown in **Figure 2A**, during the pre-COVID period, influenza positivity rates remained high, with an overall positivity rate of 25.29%. Compared with the pre-pandemic period, positivity rates during the pandemic (12.07%) decreased significantly (P< 0.001). See **Supplementary Tables 3, 4**. Entering the post-COVID period, although the positivity rate (12.42%) showed some recovery, it remained below pre-pandemic levels. This indicates that despite the potential lifting of NPIs, the epidemic intensity of this pathogen has not returned to pre-pandemic baseline levels. The pre-pandemic baseline seasonal patterns were further analyzed. Data analysis revealed highly significant seasonal variations (P< 0.001). Winter was the peak season for this pathogen, with a median positivity rate of 47.6%. Spring followed, with a median positivity rate of 1.9%, but with greater variability. Summer and autumn exhibited extremely low positivity rates, with a median of 0.0%. *Post hoc* comparisons revealed significantly higher positivity rates in winter and spring than in summer and autumn (P< 0.001), as shown in **Supplementary Table 5**, confirming the pathogen’s characteristic winter-spring epidemic pattern (**Figure 2B**). The time-series trends visually illustrated the dynamic process of epidemic fluctuations. From 2016 to 2019, positivity rates exhibited regular annual cyclical fluctuations, with peaks predominantly occurring in winter and spring. However, following the onset of the COVID-19 pandemic in early 2020, this annual cycle was disrupted. From 2020 to 2022, only sporadic fluctuations were observed, with peak intensities significantly lower than the pre-pandemic levels. Entering 2023, the re-emergence of periodic fluctuations indicated a gradual resumption of pathogen transmission activity. However, the amplitude and morphology of the epidemic curve exhibit certain heterogeneities compared to pre-pandemic patterns. As shown in **Figure 2C**, to further investigate the stability of seasonal characteristics, we conducted a stratified analysis of seasonal positivity rates across various periods. As shown in **Figure 2D**, the results indicated that while winter consistently exhibited the highest positivity rates, both the median positivity rate and range during the winter months were markedly lower during the pandemic and post-pandemic periods than in the pre-pandemic period. Concurrently, the epidemic intensity during spring was significantly suppressed both during and after the pandemic. Notably, although summer and autumn typically represent low-incidence periods, an abnormal increase in positivity rates was observed at some sampling sites during autumn in the post-COVID phase. This suggests that the epidemic seasonality of this pathogen may have shifted slightly, or that non-seasonal sporadic activity increased in the post-pandemic period.

### 3.4 Impact of environmental factors on influenza incidence

**Figure 3:**
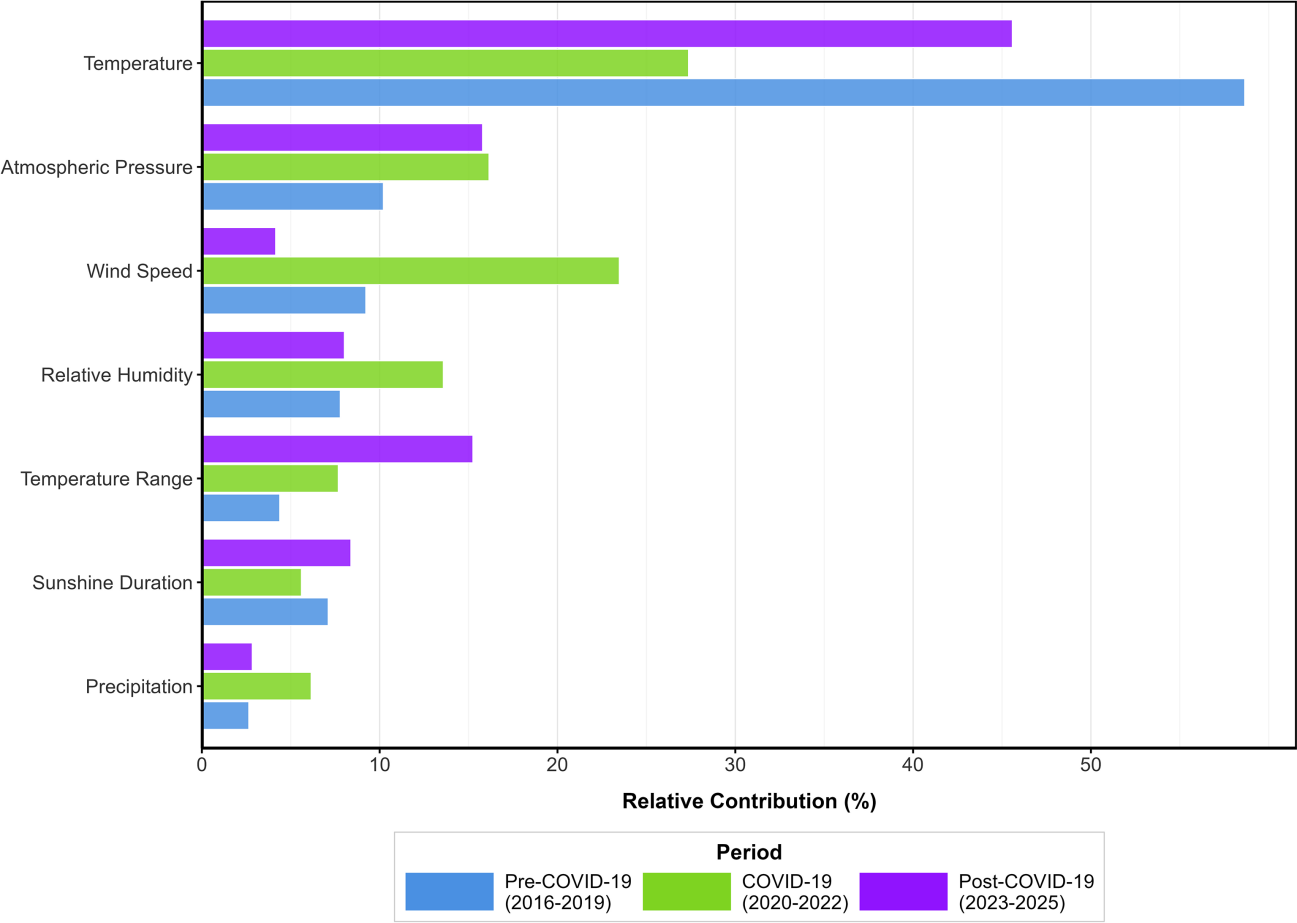
Relative contribution of environmental factors to influenza comparative analysis across COVID−19 pandemic periods.

The relative contributions of meteorological factors to influenza virus circulation exhibited marked heterogeneity across the three epidemiological phases (**Supplementary Table 6**; **Figure 3**). Temperature dominated as the primary meteorological driver throughout all periods; however, its relative contribution declined substantially during the pandemic phase (pre-pandemic: 58.65%; pandemic: 27.37%) before partially recovering in the post-pandemic period (45.58%), suggesting that NPI-induced behavioral modifications may have transiently attenuated the temperature–transmission pathway.

Several secondary meteorological factors demonstrated notably divergent trajectories during the pandemic. Wind speed exhibited the most pronounced relative increase, rising from 9.22% pre-pandemic to 23.48% during the pandemic (an increase of 14.26 percentage points), temporarily ranking second among all meteorological predictors. Similarly, atmospheric pressure (10.21% → 16.15%) and relative humidity (7.79% → 13.58%) both showed elevated relative contributions during this period, collectively accounting for 29.73% of the total meteorological signal during the pandemic phase compared to 17.00% in the pre-pandemic phase. These shifts may reflect structural changes in the environment–behavior–transmission system operating under NPI-modified conditions. However, given the substantially limited explanatory power of the SARIMAX model during the pandemic phase (R^2^ = 0.037), these redistribution patterns should be interpreted as exploratory signals rather than definitive evidence of altered causal pathways.

In the post-pandemic phase, the contribution of temperature partially recovered (45.58%), whereas the temperature range demonstrated a disproportionate increase relative to pre-pandemic levels (pre-pandemic: 4.38%; post-pandemic: 15.24%; Δ = +10.86 percentage points), emerging as the second-largest meteorological contributor in this period. This pattern suggests that diurnal temperature variability may have become a relatively more influential determinant of influenza transmission in the post-pandemic context, potentially reflecting shifts in population susceptibility or circulating strain composition following the disruption of normal seasonal exposure patterns. Conversely, wind speed returned to comparatively modest levels (4.15%), suggesting that its elevated pandemic-period contribution was transient and likely NPI-contingent. Nevertheless, given the constrained model fit during the post-pandemic period (R^2^ = 0.067), this interpretation remains tentative pending independent replication.

Collectively, these findings reveal a coherent pattern of meteorological signal redistribution across pandemic phases, with temperature consistently serving as the dominant driver, while secondary factors, particularly wind speed, atmospheric pressure, and temperature range, exhibit phase-dependent fluctuations consistent with NPI-mediated disruption of established transmission pathways. These preliminary findings warrant independent validation through prospective studies that incorporate behavioral, immunological, and virological covariates (**Supplementary Table 6**).

**Figure 4:**
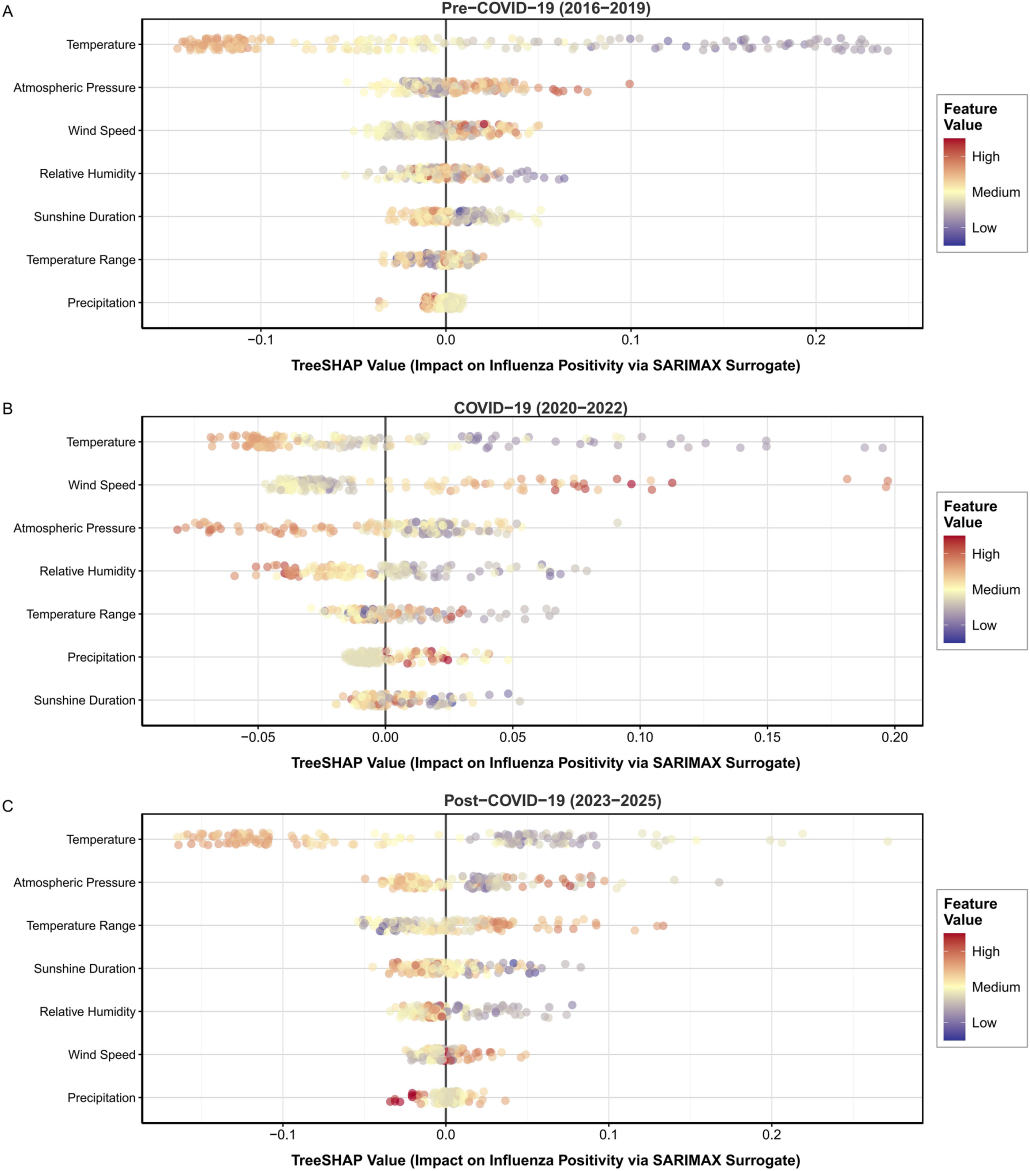
SHAP analysis: environmental factors impact on influenza across COVID−19 periods. Each dot represents one sample; color indicates standardized feature value. **(A)** the pre-COVID-19 period (2016–2019), **(B)** the COVID-19 period (2020–2022), and **(C)** the post-COVID-19 period (2023–2025). (blue = low; red = high).

To comprehensively reveal the dynamic changes in the environmental factors influencing influenza transmission, we employed an interpretability analysis that combined the SARIMAX model with the TreeSHAP proxy model. As shown in **Figures 4A–C**, the results indicate that temperature consistently ranks among the features with the widest TreeSHAP value distribution range and most significant influence, exhibiting highly consistent directionality: while high-temperature observations (yellow to red scatter points) were markedly skewed toward the left side of the X-axis (negative SHAP value range, –0.10 to 0), indicating that higher temperatures exert an inhibitory effect on influenza positivity rates. This pattern remains highly consistent across **Figures 4A–C**, confirming that “low temperatures promote, while warming suppresses” influenza transmission as a core environmental effect spanning the pre-COVID, COVID-19, and post-COVID phases. Pandemic-related interventions did not alter the fundamental biological link between temperature and influenza spread. The TreeSHAP distributions for atmospheric pressure and wind speed also exhibited distinct temporal differences across the three phases. Their TreeSHAP values clustered relatively tightly, with most points concentrated near zero, suggesting weaker marginal effects on influenza positivity rates than temperature and less pronounced directionality. As shown in **Figure 4A**, the wind speed scatter plot exhibits significant expansion in both the x- and y-directions. Observations of high wind speeds (red points) predominantly cluster in the positive TreeSHAP range (approximately 0–0.15), whereas low wind speed points (blue) cluster more heavily in the negative range. This suggests the increased importance of ventilation and aerosol transmission dynamics under non-pharmaceutical intervention conditions. Concurrently, the TreeSHAP distribution for atmospheric pressure exhibited a “wider point cloud distribution” than that at pre-pandemic levels. Scatter points under high-pressure conditions leaned toward positive values, suggesting that specific high-pressure weather patterns may favor influenza transmission or the occurrence of medical consultations and testing. As shown in **Figure 4B**, the TreeSHAP distributions for wind speed and atmospheric pressure exhibited slightly reduced amplitudes compared to the COVID-19 period, indicating a general weakening of their overall impact intensity. However, their effects remained stronger than pre-pandemic levels, suggesting that post-pandemic behavioral and environmental interaction patterns may not have fully reverted to pre-pandemic conditions. As shown in **Figure 4C**, relative humidity exhibited moderate and slightly nonlinear effects in all three phases. During the COVID-19 period, high humidity tended toward negative TreeSHAP values, suggesting that it may have a limited inhibitory effect on influenza transmission. The TreeSHAP values for sunshine duration and precipitation remained close to zero across all phases, indicating that their independent contribution to predicting influenza positivity rates was minimal. The TreeSHAP distribution for the temperature range broadened slightly in the post-COVID phase, showing some association between higher ranges and increased positivity rates, although its impact remained markedly weaker than the absolute temperature. Overall, TreeSHAP results indicate that temperature-related effects maintained high consistency across all three phases, while the environmental driver structure underwent reshaping during COVID-19, with circulation-related factors such as wind speed and atmospheric pressure gaining importance. In the post-pandemic phase, this structure partially reverted to pre-pandemic patterns while retaining new environmental response characteristics, such as heightened sensitivity to wind speed and temperature differences. This suggests that under the combined influence of behavioral changes and environmental adaptation, the dependence of influenza on external meteorological conditions is evolving toward a new equilibrium state.

## 4 Discussion

This study systematically assessed influenza circulation patterns before and after the COVID-19 pandemic and its reshaping effects on environmental drivers, based on continuous monitoring data from Jiuquan, China, spanning 2016–2025. The results indicate that influenza in this region exhibits a typical northern hemisphere pattern of high incidence during winter and spring. COVID-19-related non-pharmaceutical interventions led to an unprecedented “low circulation” during 2020–2022. Although influenza activity rebounded significantly in the post-pandemic period, it displayed characteristics that were distinct from the pre-pandemic era in terms of epidemic intensity, subtype composition, and environmental sensitivity. Prior to the pandemic, influenza epidemics in Jiuquan exhibited typical seasonal and subtype-specific patterns. The peak season occurred from winter to the following spring and was characterized by distinct peaks, predominantly driven by a single subtype. A/H3N2 was the predominant subtype, whereas B/Victoria maintained relatively stable circulation. B/Yamagata was detected sporadically, and A/H1N1 caused intermittent outbreaks. This pattern aligns with historical influenza circulation patterns in the temperate regions of the Northern Hemisphere (Zhang, et al., 2025), indicating that Jiuquan exhibited typical “conventional influenza ecology” before the COVID-19 pandemic. However, starting in early 2020, following the implementation of NPIs, we observed the near-complete “disappearance” of all influenza subtypes during 2020–2021 in Jiuquan. Epidemiologically, this manifested as a sharp decline in weekly positivity rates to<5% beginning in March 2020, followed by 78 consecutive weeks from April 2020 to October 2021 without any traditional winter peaks, with weekly case counts persistently maintained at historically low levels (0–5 cases). This prolonged and profound interruption of influenza circulation is exceptionally rare in the historical surveillance records. This aligns closely with the “near disappearance of influenza” observed in multiple regions globally during COVID-19 containment efforts (Sullivan et al., 2020; Song et al., 2022), further supporting the notion that NPIs exert a non-specific inhibitory effect on respiratory virus transmission (Zhao et al., 2024). Notably, during the 2021–2022 winter season in Jiuquan, only a low-level resurgence of B/Victoria was observed, whereas influenza A was almost completely absent. This suggests different sensitivities to NPIs and altered population behaviors among the subtypes. Lineages with weaker or moderate transmissibility may face greater difficulty in reestablishing sustained transmission chains under intense intervention, resonating with global discussions on the “candidate disappearance” of B/Yamagata in some regions (Paget et al., 2022). Following adjustments to national prevention strategies after 2023, the epidemiological landscape underwent a significant transformation, and the 2023–2024 influenza season featured a distinct and intense winter epidemic peak, with a marked rebound in influenza positivity rates. Epidemic intensity notably “escaped” the suppressed state observed between 2020 and 2022. A/H3N2 re-emerged as the dominant subtype, whereas A/H1N1 and B/Victoria were circulating concurrently. Importantly, the duration of positivity rates exceeding 10% increased from a pre-pandemic average of 7 weeks to 11 weeks post-pandemic. This suggests that under prolonged low exposure, declining population immunity and the accumulation of susceptible individuals—the so-called “immunity debt” effect—may be concentrated in the post-pandemic phase (Wu et al., 2024). The winter 2024–2025 peak pattern further evolved, with alternating dominance between A/H3N2 and A/H1N1, indicating ongoing niche redistribution and competition among subtypes. Meanwhile, B/Yamagata failed to generate a distinct epidemic peak throughout the post-pandemic period, supporting the hypothesis that it may be in a state of “persistent occultation” or even “functional extinction” (Caini et al., 2024).

This study systematically compared the distribution and temporal variations in weekly positivity rates across three periods and four seasons from 2016 to 2025. Significant differences were observed in the positivity rates among the three study periods (p = 0.0147). The overall positivity rate in the pre-COVID period was 25.29%, declining to 12.07% during the pandemic (COVID-19). Although it rebounded to 12.42% in the post-pandemic phase, it remained significantly lower than the pre-pandemic level (P< 0.001). This indicates that influenza did not immediately return to its pre-pandemic baseline even after control measures were lifted, consistent with the findings of Chen et al (Chen et al., 2024). Its overall epidemic potential may have been reshaped by multiple factors, including changes in population immunity, pathogen lineage composition, and behavioral patterns. From a seasonal perspective, the pre-pandemic data exhibited pronounced seasonal variations. The median positivity rate reached 47.6% in winter, followed by spring with greater variability, while summer and autumn exhibited near-absent or silent transmission, clearly delineating the pathogen’s typical winter-spring peak pattern. During the COVID-19 period, this annual cycle was nearly completely disrupted due to the lockdown. The time-series curves show that the regular peaks and troughs observed from 2016 to 2019 were reduced to sporadic fluctuations with extremely low peaks from 2020 to 2022. The post-COVID phase introduced new annual cyclical fluctuations, indicating a gradual resumption of influenza transmission. However, the amplitude, peak width, and timing of peaks now differ significantly from pre-pandemic patterns: while winter remains the primary peak season, partial anomalous elevations appear in autumn, suggesting a potential shift from the traditional concentrated winter-spring pattern toward a more “extended” or “sporadically enhanced” model. This seasonal “loosening” may be associated with altered population mobility patterns, abnormal climate fluctuations, and host immune interference caused by the co-circulation of other respiratory viruses. Further multi-pathogen, multi-region collaborative analyses are needed to validate these observations.

When assessing the impact of environmental factors on influenza positivity rates, this study employed a SARIMAX model incorporating exogenous variables and conducted an interpretability analysis using the TreeSHAP proxy model. It is important to note that the goodness-of-fit of the models differed significantly across phases: the SARIMAX model demonstrated moderate fit in the pre-COVID phase (R^2^ = 0.5189, RMSE = 0.1806), whereas the fit was poor in both the COVID-19 phase (R^2^ = 0.0373, RMSE = 0.1198) and the post-COVID phase (R^2^ = 0.0671, RMSE = 0.0833), which exhibited lower R^2^ values, explaining only a small proportion of the variation in positivity rates. This indicates that during the pandemic and its aftermath, factors beyond conventional meteorological variables, such as NPIs, changes in healthcare-seeking behavior, viral lineage shifts, and other unobserved covariates, exerted a more significant influence on influenza activity. Consequently, time series models relying solely on meteorological variables have limited explanatory power regarding the epidemic intensity. Concurrently, the TreeSHAP proxy model demonstrated high fidelity to SARIMAX fitted values across all three phases (R^2^ = 0.8121–0.8628), suggesting stable decomposition of the relative contributions from meteorological factors given the SARIMAX output. Therefore, the SARIMAX+TreeSHAP results in this study should be interpreted as an explanatory characterization of the “environmental driving structure and its phased rearrangement,” rather than as precise predictions of positivity rate variations or quantitative estimates of causal effects from individual meteorological factors. Under this premise, the environmental relative contribution analysis revealed that during the pre-COVID phase, influenza epidemics were primarily driven by temperature, with wind speed playing a relatively minor role. During the COVID-19 phase, the relative contribution of wind speed surged from approximately 9.22% to 23.48%, becoming the second most critical environmental factor after temperature. The weights of the relative humidity and atmospheric pressure also increased from 7.79% and 10.21% to 13.58% and 16.15%, respectively. In the post-COVID phase, temperature partially regained its dominant role, while the contribution of temperature differences notably surpassed pre-pandemic levels (15.24% vs. 4.38%), suggesting heightened potential impacts of temperature variability on influenza transmission in the post-pandemic era. TreeSHAP scatter plots further corroborate these structural shifts through directional and non-linear patterns. Across all three phases, the temperature TreeSHAP value distribution consistently exhibited highly consistent patterns: higher-temperature observation points (warm-colored scatter points) predominantly occupied the negative SHAP value range (–0.10 to 0), indicating that high temperatures inhibit influenza transmission. This directionality was particularly evident during the pre-COVID phase, with moderate model fit, offering localized support for established biological and epidemiological understanding that “lower temperatures favor influenza transmission.” (Hanzlíková et al., 2025; Shang et al., 2026; Amendolara et al., 2026; Shi et al., 2026). While this pattern persisted in the COVID-19 and post-COVID phases with a lower model fit, it should be interpreted more as a trend signal consistent with prior patterns rather than strong causal evidence (Shi et al., 2026). In contrast, the TreeSHAP distributions for wind speed and atmospheric pressure noticeably “widened” during the COVID-19 phase, with data points under high-wind and high-pressure conditions tending toward positive TreeSHAP values. This suggests that environmental factors related to ventilation, aerodynamics, and circulation patterns gained relative weight in the model under conditions of strong interventions and low incidence. However, given the near-zero R^2^ during this phase, this “weight increase” likely reflects the model capturing residual correlations from limited fluctuations amid a weakened overall signal, rather than indicating that wind speed or pressure became dominant drivers during the pandemic. In the post-COVID phase, this expansion partially retracted but remained above pre-pandemic levels, suggesting that the environmental-behavioral-transmission system had not fully returned to its original equilibrium. The TreeSHAP values for relative humidity, sunshine duration, and precipitation remained near zero across all three phases, indicating limited independent contributions to positive rate variation. This may partly reflect the relatively constrained annual variability of relative humidity, precipitation, and sunshine duration in Jiuquan’s dry, cold inland region, where their marginal effects are easily overshadowed by dominant variables such as temperature and wind speed. This also suggests that a more comprehensive understanding of the environmental drivers of influenza requires the incorporation of non-meteorological factors, such as air pollution, indoor environments, and population mobility, into analytical frameworks. Overall, we adopted a relatively conservative interpretive stance toward the SARIMAX–TreeSHAP results: Temperature-related effects showed highly consistent directionality across all three phases, suggesting robust conclusions. while the increased significance of factors like wind speed, atmospheric pressure, and temperature differences during the COVID-19 and post-COVID phases is better understood as “structural signals” reflecting complex reorganization within the environment-behavior-transmission system under intervention contexts. These findings warrant further validation using multi-regional data, richer covariates, and causal inference methods in the future.

This study systematically characterized the reshaping of influenza ecology before and after the COVID-19 pandemic across three complementary dimensions—epidemiological patterns, seasonal characteristics, and environmental drivers—offering multifaceted insights into the public health implications. First, while high-intensity, prolonged NPIs significantly suppressed the transmission of influenza and other respiratory pathogens (Lenglart et al., 2025; Liang et al., 2025), they also drastically reduced opportunities for natural exposure. This approach may have flattened epidemic curves in the short term, but simultaneously accumulated “immunological debt (Li et al., 2026)” through waning immune protection and unexposed newborn and pediatric cohorts. Following the withdrawal of control measures, this immune debt may manifest as more concentrated, intense epidemic peaks and prolonged high-prevalence periods, as preliminarily observed during the 2023–2024 influenza season in this study. When addressing future pandemics of emerging infectious diseases, it is essential to develop high-intensity NPIs while simultaneously assessing their potential long-term impacts on the burden of other respiratory diseases. Proactive planning is required for vaccination strategies, protection of vulnerable populations, and allocation of healthcare resources. Second, while seasonal patterns partially recovered in the post-COVID era, new characteristics such as heightened sporadic activity in autumn and prolonged epidemic peaks emerged, underscoring that influenza risk cannot be simplistically viewed as a “winter problem.” Against the backdrop of climate change, accelerated urbanization, and shifting population mobility patterns, it is essential to integrate high-precision meteorological data, population behavior information, and multi-pathogen surveillance results into a unified platform. This will enable dynamic updates to regionalized influenza early warning systems and allow targeted interventions during high-risk periods and among vulnerable populations. Third, model analysis indicated that temperature consistently functioned as a primary environmental signal for influenza transmission across all phases. In contrast, the temporal variability in the relative importance of wind speed, atmospheric pressure, and diurnal temperature range underscored the potential contributions of ventilation conditions and extreme weather events to transmission dynamics. It should be noted that these inferences are based on environmental variable weighting analysis derived from the SARIMAX + TreeSHAP framework; however, the SARIMAX model’s overall explanatory power remains limited, and these relationships should be interpreted as associative rather than causal. Accordingly, at the levels of urban planning and public facility management, it is imperative to develop adaptive respiratory disease prevention and control systems suited to the post-pandemic era through optimized seasonal ventilation strategies and minimized exposure risks during extreme weather conditions.

This study had several limitations. First, the data were derived from sentinel surveillance in a single region, which was potentially influenced by healthcare-seeking behavior, utilization of medical services, and adjustments to monitoring strategies. Second, environmental factors were limited to conventional meteorological variables, failing to incorporate sociophysical factors such as air pollution, population mobility, and indoor environments, which may have underestimated the combined effects of multisource exposures. Third, although the SARIMAX+TreeSHAP method offers advantages in time-dependent control and interpretability, the resulting associations are based on observational data, making it difficult to directly infer causality at the individual level.

In summary, this study preliminarily observed that the COVID-19 pandemic in Jiuquan, China, not only disrupted influenza transmission in the short term but also reshaped its long-term epidemiological characteristics. Influenza has gradually transitioned from a complex pattern traditionally co-regulated by multiple environmental factors and accompanied by subtype alternation to a new phase primarily influenced by temperature, with simplified overall environmental drivers. This shift underscores the dynamic evolution of infectious disease ecology. Future research should conduct multi-center, multi-climate-zone collaborative analyses to continuously monitor changes in the interactive network among social behaviors, environmental conditions, viral evolution, and host immunity. This will enable timely responses to potential future epidemiological shifts while providing more robust evidence-based support for the comprehensive prevention and control of respiratory infectious diseases in the post-pandemic era.

## Data Availability

The original contributions presented in the study are included in the article/**Supplementary Material**. Further inquiries can be directed to the corresponding authors.
