# Supplementary material for: Three-stage interpretability analysis of influenza virus and meteorological correlation in Jiuquan City, 2016–2025: SARIMAX + TreeSHAP
Source: Front Cell Infect Microbiol. 2026 Jun 10;16:1791100. doi: 10.3389/fcimb.2026.1791100 (PMC13290768; doi:10.3389/fcimb.2026.1791100)
Supplement: Supplementary file 1 [file Table1.docx]

Table S1 Evaluation Metrics for SARIMAX + TreeSHAP Surrogates Model

| Period | *R²* | *RMSE* | *Surrogate Fidelity* | *Model* | *AICc* | *Log-Likelihood* |
| --- | --- | --- | --- | --- | --- | --- |
| Pre-COVID | 0.5189 | 0.1806 | 0.8628 | ARIMA(1,0,3)(0,0,0)[52] | -245.64 | 135.85 |
| COVID | 0.0373 | 0.1198 | 0.8121 | ARIMA(1,1,3)(0,0,0)[52] | -270.19 | 148.53 |
| Post-COVID | 0.0671 | 0.0833 | 0.8363 | ARIMA(0,1,0)(0,0,0)[52] | -154.36 | 85.81 |

Table S2 Evaluation Metrics for SARIMAX + TreeSHAP Surrogates Model

Performance metrics of phase-stratified and NPI-excluded SARIMAX models for meteorological-influenza association analysis across epidemiological periods

| Period | *R²* | *RMSE* | *Surrogate Fidelity* | *Model* | *AICc* |
| --- | --- | --- | --- | --- | --- |
| Pre-COVID | 0.5189 | 0.1806 | 0.8628 | ARIMA(1,0,3)(0,0,0)[52] | -245.64 |
| COVID | 0.0373 | 0.1198 | 0.8121 | ARIMA(1,1,3)(0,0,0)[52] | -270.19 |
| Post-COVID | 0.0671 | 0.0833 | 0.8363 | ARIMA(0,1,0)(0,0,0)[52] | -154.36 |
| Pre+Post | 0.2782 | 0.1444 | 0.7878 | ARIMA(2,0,2)(0,0,0)[52] | -354.422 |

Table S3 Differential Analysis of Positive Rates of Influenza Across Distinct Periods

| Characteristici | Postive rate | *P*-Value |
| --- | --- | --- |
| Period |  | *P*=0.0147 |
| Pre-COVID | 25.29% |  |
| COVID | 12.07% |  |
| Post-COVID | 12.42% |  |

*Noted: Three-group comparisons performed using Kruskal-Wallis test ; pairwise comparisons using the Dunn test, and correct for multiple comparisons using the Bonferroni method., with statistical significance set at P<0.05.*

Table S4 Differential Analysis of Positive Rates of Influenza Across Distinct Periods

| Period | *Z* | *P*-Value |
| --- | --- | --- |
| Pre- vs COVID | -2.846 | ＜0.05 |
| Pre- vs Post- | -0.693 | 0.732281 |
| COVID vs Post- | -2.025 | 0.064324 |

*Noted: Three-group comparisons performed using Kruskal-Wallis test ; pairwise comparisons using the Dunn test, and correct for multiple comparisons using the Bonferroni method., with statistical significance set at P<0.05.*

Table S5 Differential Analysis of Positive Rates of Influenza Across Distinct Seasons

| Season | *Z* | *P*-Value |
| --- | --- | --- |
| Spring vs Summer | 4.259 | ＜0.001 |
| Spring vs Autumn | -2.441 | <0.05 |
| Spring vs Winter | -4.622 | ＜0.001 |
| Summer vs Autumn | 1.766 | 0.2321 |
| Summer vs Winter | -8.878 | ＜0.001 |
| Autumn vs Winter | -7.004 | ＜0.001 |

*Noted: Three-group comparisons performed using Kruskal-Wallis test ; pairwise comparisons using the Dunn test, and correct for multiple comparisons using the Bonferroni method., with statistical significance set at P<0.05.*

Table S6 Contribution (%) of Environmental Factors across Different Periods for Influenza

| Environmental factors | Pre-COVID | | COVID-19 | Post-COVID |
| --- | --- | --- | --- | --- |
| Temperature | 58.65 | 27.37 | | 45.58 |
| Atmospheric Pressure | 10.21 | 16.15 | | 15.79 |
| Relative Humidity | 7.79 | 13.58 | | 8.02 |
| Sunshine Duration | 7.11 | 5.60 | | 8.38 |
| Precipitation | 2.64% | 6.15 | | 2.84 |
| Temperature Range | 4.38 | 7.67 | | 15.24 |
| Wind Speed | 9.22 | 23.48 | | 4.15 |
